# Heavy Menstrual Bleeding in the Gynecology Clinic: A Call for Awareness and Standardized Screening for Underlying Bleeding Disorders

**DOI:** 10.1097/OGX.0000000000001514

**Published:** 2026-05-07

**Authors:** Silvia Luijten, Laurens Nieuwenhuizen, Karin P.M. van Galen, Marlies Y. Bongers, Peggy M.A.J. Geomini, Jaklien C. Leemans

**Affiliations:** a Department of Hematology, Máxima Medical Center, Veldhoven; b Grow, Research institute for oncology and reproduction, Maastricht University, Maastricht; c Department of Hematology, van Creveld Clinic, University Medical Center Utrecht; d Department of Obstetrics and Gynecology, Máxima Medical Center, Veldhoven, The Netherlands

**Keywords:** heavy menstrual bleeding, bleeding disorders, von Willebrand disease, PBAC, Philipp tool, self-BAT

## Abstract

**Importance:**

Heavy menstrual bleeding (HMB) is a common problem existing in nearly 30% of women worldwide. An underlying bleeding disorder has been identified as a cause of HMB in a significant proportion of women seeking gynecological care for HMB. In practice, however, there is a significant underdiagnosis of bleeding disorders in these patients, highlighting a need for improvement in awareness and detection.

**Objective:**

To present a comprehensive review of the literature on HMB and bleeding disorders and to increase awareness of their interrelation. In addition, to propose a standardized diagnostic approach to help identify which women with HMB may have an underlying bleeding disorder and require further hemostatic evaluation.

**Evidence Acquisition:**

A literature review was performed using PubMed to identify relevant primary research and review articles on the topic of HMB and bleeding disorders in English. This was supplemented by manual reference searches and review of relevant guidelines in English and Dutch.

**Results:**

A significant proportion (10% to as much as 62%) of women with HMB have an underlying bleeding disorder, yet many such disorders go unrecognized. Various screening instruments, particularly the validated PBAC, Philipp tool or self-BAT, can help distinguish between HMB with or without a suspected bleeding disorder, and indicate who needs additional hemostatic evaluation.

**Conclusions and Relevance:**

An underlying bleeding disorder is far more frequent in HMB than currently appreciated. Gynecologists should be aware of this and be familiar with screening instruments and a diagnostic workflow for bleeding disorders. Multidisciplinary collaboration between gynecology and hematology is crucial for accurately diagnosing and managing potential bleeding disorders in women with HMB.

## LEARNING OBJECTIVES

After completing this activity, the learner will be better able to:• Describe factors that are suspected of an underlying bleeding disorder in HMB patients• Explain how to adequately screen for underlying bleeding disorders in HMB patients

**Target audience:** Obstetricians and gynecologists, family physicians

Heavy menstrual bleeding (HMB) is common in the general population, affecting 30% of women, and results in frequent health care consultation.^1,2^ HMB has a profound impact on quality of life, affecting physical, emotional, and social quality of life while also imposing a high economic burden.^3^ Women with HMB, who also have bleeding disorders, often find it challenging to fully participate in school, work, athletic, and social activities, and experience a diminished quality of life.^4,5^ Heavy blood loss can lead to significant decreased iron and hemoglobin levels. As a result, HMB is the most common cause of iron deficiency anemia in healthy, fertile women.^6^ Iron deficiency anemia is associated with physical^7^ and cognitive impairment^8,9^ and a reduced health-related quality of life.^10^

## CAUSES OF HMB

HMB is often a multifactorial problem and can be caused by uterine disorders and/or other causes, like coagulopathy. To help health care providers classify the causes of abnormal uterine bleeding, like HMB, the International Federation of Gynecology and Obstetrics (FIGO) created a categorization system.^11^ Causes of abnormal uterine bleeding in this system are polyp, adenomyosis, leiomyoma, malignancy, coagulopathy, ovulatory dysfunction, endometrial, iatrogenic, and not otherwise classified; summarized as PALM-COEIN.^12^ Coagulopathy is defined as a condition in which the blood’s ability to form clots is impaired. The blood coagulation mechanism is complex, requiring a cascade of activating platelets, proteins and clotting factors. In people with a bleeding disorder, the coagulation process is disrupted. This can be due to a wide range of possible causes, including platelet and or clotting factor deficiencies and or dysfunction. HMB resulting from an underlying bleeding disorder is categorized in the PALM-COEIN system as coagulopathy.

## BLEEDING DISORDERS AS A CAUSE OF HMB

Gynecologists traditionally focus on identifying anatomic disorders as potential causes of HMB. However, there is an emergent recognition of the importance of considering coagulopathy or underlying bleeding disorders in this patient population. In general, women with bleeding disorders are underrecognized and underdiagnosed.^13^ This is also evident in cases of HMB, where clinicians rarely consider a bleeding disorder in their differential diagnosis,^14,15^ despite their high prevalence (see Recommendation on how to screen individuals with HMB for further hemostatic testing for bleeding disorders section). There may be several reasons why underlying bleeding disorders are left undiagnosed in HMB. First, the severity of HMB can be overlooked. This can occur if specific HMB measurement tools are not used to obtain an objective quantification of menstrual blood loss.^16^ Second, without evident concurrent bleeding symptoms, general practitioners and gynecologists may overlook an underlying bleeding disorder, especially when patients have not yet encountered significant bleeding challenges.^17^ Third, concurrent bleeding symptoms are often neither inquired about nor thoroughly examined during consultations. This leads to missed opportunities for diagnosis. Moreover, even when a gynecological cause is identified, a coagulation disorder may not be considered, even though both can coexist.^18^ Fourth, limited access to specialized laboratory testing or referral to a hematologist with expertise in bleeding disorders also poses a barrier to a timely diagnosis.^19^ This underscores a critical gap in knowledge and awareness within the gynecological community. Diagnosing an underlying bleeding disorder is not only important for the treatment of HMB, but also ensures proactive management of bleeding risks during future events like trauma, delivery, or surgery. Hematologists must also pay attention to women’s menstrual bleeding complaints in their bleeding disorder population. If hematologists and gynecologists are aware of the underlying causes of HMB, more effective and personalized treatment is possible. An integrated understanding of both anatomical and nonanatomic factors should be the groundwork in the collaborative efforts of gynecologists and hematologists in the care of HMB.

## AIM AND LITERATURE ACQUISITION

This review summarizes the literature on HMB and bleeding disorders and highlights the need for greater awareness of their connection. It proposes a standardized workup for women with HMB that includes consideration of underlying bleeding disorders. A literature review was performed with the help of a librarian using PubMed to identify relevant primary research and review articles on the topic of HMB and bleeding disorders in English, using the following terms: “Menorrhagia” OR “Heavy Menstrual Bleeding*” OR “Hypermenorrh*” OR “menorrhag*” OR “Heavy Period*” OR “abnormal menstrual bleed*” OR “heavy menstruat*” AND “Bleeding assessment tool*” OR “Bleeding score*“ OR “blood coagulation test*“ OR “ bleeding scale*“ OR “ISTH-BAT*” OR “partial thromboplastin time*” OR “factor VIII activit*” OR “von-Willebrand-factor antigen*“ OR “factor XI*“ OR “trombocyt*” OR “Pt aptt” OR “thrombin time” OR “Fibrinogen*” OR “platelet function*” OR “Factor ii*” OR “factor five” OR “factor vi*” OR “factor X*” OR “factor ix*.” This search was supplemented by a manual search of relevant references and guidelines in English and Dutch.^20–22^

## HMB DEFINITION AND MEASURING BLOOD LOSS

There are different definitions of HMB worldwide. Classically, HMB was defined in 1966 as menstrual blood loss exceeding >80 mL blood loss per cycle.^23^ The FIGO definition nowadays is: excessive menstrual blood loss that interferes with a woman’s physical, emotional, social and material quality of life.^24^ The National Institute for Health and Care Excellence in the United Kingdom defines HMB in the same way as the FIGO. However, they footnote that it may occur alone or in combination with other symptoms and possibly with menstrual blood loss of <80 mL.^21^ Despite this, the definition of HMB remains inconsistent. An online survey among gynecologists and general practitioners treating HMB in 10 countries shows a statistically significant difference in the definition of HMB. Most health care practitioners consider more than 7 days of bleeding abnormal.^25^ To accurately measure >80 mL blood loss per cycle is difficult. An objective classical method, the alkalin-hematin method, originally proposed by Hallberg et al,^23^ and later modified by van Eijkeren et al^26^ involves chemically measuring the blood content of used sanitary products. However, this method is laborious, time-consuming and not user-friendly for patients. In addition, the number of sanitary pads and tampons does not correlate with the amount of menstrual blood loss.^27^ Recently, a study found considerable variability in red blood cell volume capacity of newer menstrual products like menstrual cups, discs, and period underwear. This underscores the importance of asking about the specific type of menstrual products used and understanding their capacity for accurately measuring blood loss.^28^ Also, upcoming apps like the Menstruation Education Calendar (MEK-APP) offer tools for measuring menstrual abnormalities.^29^ Currently, the most common measuring instrument is the Pictorial Bleeding Assessment Chart (this is a self-assessed and validated tool developed to visually assess the amount of menstrual blood loss). It uses the saturation of sanitary products as a guide. A systematic review on the use of PBAC reported varying sensitivity (58% to 99%) and specificity (7.5% to 89%) for the diagnosis of blood loss >80 mL. In addition, the sensitivity for the diagnosis of self-perceived HMB was 78.5% and the specificity was 75.8%.^30^

## COLLABORATION BETWEEN GYNECOLOGIST, HEMATOLOGIST, AND NURSE SPECIALIST

Inherited bleeding disorders often go underdiagnosed in women with HMB.^14,31^ This underscores the need for increased awareness and collaboration between gynecology and hematology. In our ongoing nationwide study among Dutch hematologists and gynecologists, we found that gynecologists pay limited attention to underlying bleeding disorders (S. Luijten et al., 2026, unpublished data). In this study, 149 gynecologists and 62 hematologists completed an online survey to evaluate the awareness and diagnostic workup regarding underlying bleeding disorders in patients with HMB. Profound differences are seen in referrals by gynecologists: the majority rarely refer women with HMB to the hematologist. Only a small percentage often make such referrals. The presence of multidisciplinary team meetings and workup protocols for bleeding disorders also varies widely in this ongoing study. Given that the assessment of HMB and underlying bleeding disorders is complex and needs more attention, the involvement of collaborative multidisciplinary teams is essential. Multidisciplinary care, tailored to individual needs, is crucial for these patients.^32^ Mulders et al^33^ propose a role for the nurse specialist in coordinating care and educating both patients and the health care team. Studies show high patient satisfaction (90%) with multidisciplinary approaches involving hematologists, gynecologists, and nurse specialists.^34^ Through the incorporation of insights from both specialties, we can approach the problem of HMB with a broader perspective. Indeed, it is demonstrated that cancer multidisciplinary teams and/or clinics are associated with changes in diagnosis,^35^ management plans,^36,37^ and adherence to clinical guidelines.^34^ In 2021, European principles of care for women and girls with inherited bleeding disorders have been published to guide physicians dealing with these patients.^38^

## HEMOSTASIS

Hemostasis is the process of blood clot formation at the site of vessel injury. It is a dynamic, highly interwoven network of multiple processes that maintains a balance between coagulants and anticoagulants.^39^ The cascade requires a trigger to start the transition from fluid blood to a clot. The mechanism of coagulation involves activation, adhesion, and aggregation of platelets, along with deposition and maturation of fibrin. Hemostasis can be conceptualized as occurring in phases: first, endothelial injury leads to the formation of a platelet plug, known as primary hemostasis; next, the coagulation cascade propagates the clotting process, termed secondary hemostasis; this is followed by the termination of the clotting process. Finally, the removal of the clot through fibrinolysis.

Hemostasis in the HMBA woman’s monthly menstruation poses a great challenge to the hemostatic system. The whole menstrual cycle includes different phases. During the menstrual period, the “old” lining of the uterus is shed. Bleeding continues during the proliferation and repair of the surface epithelium of the damaged stratum functionalis. This shedding process of the lining of the uterus demands effective hemostasis to prevent excessive bleeding. Bleeding disorders impair hemostasis, which may lead to excessive or prolonged bleeding. They can result from issues in primary hemostasis (eg, von Willebrand disease, inherited platelet function disorders), secondary hemostasis (eg, hemophilia A and B, other (rare) coagulant factor deficiencies), fibrinolysis, or connective tissue and vascular formation.^40^

## PREVALENCE OF BLEEDING DISORDERS IN HMB

HMB can be the first manifestation in a woman’s life of an underlying bleeding disorder.^41^ Bleeding disorders include mostly von Willebrand disease (VWD), platelet disorders, hemophilia, bleeding disorder of unknown cause (BDUC), and, less commonly, fibrinolytic disorders or clotting factor deficiencies. The prevalence of underlying bleeding disorders in cases of HMB differs widely in the literature, ranging from at least 10%^42–45^ to 29%-62%.^18,46–51^ Several factors may contribute to these variations. First, there was mostly no strict definition of HMB in these studies. Patients were frequently included based on their attendance at clinics for HMB complaints, without standardized blood loss measurement methods. However, Miller et al,^44^ did define HMB more precisely and included only patients with a PBAC score >100. In this group, 6% of HMB patients were diagnosed with von Willebrand disease and 56% with a platelet disorder. Second, the study populations varied in age (adolescents or above 18 to 21 years old) and selection criteria. The studies predominantly included women who were referred to specialized multidisciplinary hematology-gynecology or hemophilia treatment center clinics. Third, the methodologies differed, with some studies being retrospective and others being prospective. They also utilized a wide range of coagulation tests and included various bleeding disorders. Therefore, the prevalence data of underlying bleeding disorders in HMB vary, depending on the population studied and the definitions used.

### Von Willebrand Disease (VWD) in HMB

VWD is the most common bleeding disorder and often manifests as mucosal bleeding. This condition arises from a deficiency in the quantity or quality of von Willebrand factor. This is a multimeric protein that is necessary for platelet adhesion. VWD can be secondary to a quantitative (type 1 and type 3) or qualitative (type 2) defect in Von Willebrand factor (VWF). Symptoms are most often nosebleeds, primary hemostasis problems such as easy bruising, bleeding after surgery/tooth extraction, bleeding gums, fluxus postpartum, and HMB. VWD’s clinical prevalence based solely on laboratory parameters is ~1 in 100.^52,53^ However, the number of patients with VWD and bleeding symptoms is likely closer to 1 in 1000.^52^ Although men and women are equally affected by VWD, women are more likely to have symptomatic bleeding. This is due to the additional hemostatic challenges associated with menstruation and childbirth.^54^ High-quality evidence from a systematic review of eleven studies demonstrates that 131 out of 988 patients with HMB were diagnosed with VWD. These diagnoses were made in hospital outpatient clinics (mainly gynecological) and population surveys, with a prevalence ranging from 5% to 24%.^45^ More recently, Alaqzam et al^49^ tested 73 adolescents with HMB attending a multidisciplinary hematology-adolescent gynecology clinic. They found that 36% had a type of VWD. Dilley et al^42^ conducted a prospective study in HMB patients among members of a health maintenance organization and found a 10.7% prevalence of VWD. A retrospective review of 124 adolescents with HMB at a tertiary gynecology clinic showed an 11% prevalence of VWD.^43^ In 232 women with a PBAC >100 who had blood testing, 6% were diagnosed with VWD.^44^ Vo et al^47^ reported that 9% of HMB patients had VWD in a retrospective review of 105 patients, while Zia et al^46^ tested 200 HMB patients and found that 25% were diagnosed with VWD and 57% had low von Willebrand factor levels. Ultimately, Knol et al^18^ showed in a single-center prospective cohort study a 5% prevalence of VWD in HMB patients. These patients had heavy, regular periods (every 23 to 39 days) and were referred to the gynecology clinic. Those with a PBAC score <100 or known bleeding disorders were excluded.

### Platelet Disorders in HMB

Effective hemostasis requires adequate circulating numbers of functional platelets. In both qualitative and quantitative platelet disorders, bleeding signs and symptoms can occur. They can range from mild to severe, depending on the type of defect. Particularly mucocutaneous bleeding, such as epistaxis, bruising, petechial bleeding, and HMB, is specific for platelet disorders.^55^ The diagnosis of platelet function disorders requires specialized laboratory testing. For example, platelet aggregation tests and sometimes genetic testing. Platelet disorders are reported as a common cause of HMB in adolescents, with prevalence rates ranging from 8% to 56%.^43,44,46,47,49^ Lowe et al^50^ showed that half of the women presenting with HMB who did not have von Willebrand disease, were diagnosed with a platelet disorder. Two studies found that 44% to 56% of women with HMB were diagnosed with a platelet defect.^44,56^ O’Brien et al^43^ conducted a retrospective study with 124 adolescents presenting with HMB, revealing that 33% were diagnosed with a platelet disorder. The prevalence of platelet function disorders may have been underappreciated in previous studies of HMB that did not systematically evaluate platelet function.^47^ In 2020, a systematic review reported that HMB occurred in 25% of women with Bernard-Soulier syndrome and in 22% of those with Glanzmann thrombasthenia.^57^ Both conditions are rare and among the most severe inherited platelet function disorders.

### Inherited Coagulation Factor Deficiencies in HMB

The most common inherited coagulation factor deficiencies are hemophilia A (FVIII) or hemophilia B (FIX). Due to the recessive X-chromosomal inheritance pattern, females are carriers of the mutation. They typically have FVIII or IX concentrations that are approximately half of those found in healthy individuals. This should be sufficient for normal hemostasis. However, carriers may also present with low clotting factor levels within the hemophilia diagnostic range.^58^ A common misconception is that hemophilia only impacts males due to the female “carrier” status. However, female carriers of hemophilia also experience bleeding issues.

Few studies have considered the prevalence of HMB in hemophilia carriers, although HMB appears to be the most commonly reported bleeding symptom, ranging from 64% to 80%.^59,60^ Data on the prevalence of coagulation factor deficiencies in women with HMB are scarce and limited to small observational and prospective studies. These studies show a low occurrence of coagulation factor deficiencies in HMB patients.^18,51^ Besides hemophilia, rarer deficiencies of FII, FV, FVII, FX, FXI, FXIII, and fibrinolytic disorders are found. These clotting factor deficiencies are commonly referred to as rare bleeding disorders (RBD) in the literature. RBD comprises only 5% of all inherited bleeding disorders worldwide.^61^ Each of these clotting factors plays a critical role in the coagulation cascade. In 2023, a large nationwide study on rare bleeding disorders and HMB was published.^62^ It revealed that 74% of women with a coagulation factor deficiency experience HMB, as indicated by a HMB-specific International Society on Thrombosis and Hemostasis Bleeding Assessment Tool (ISTH BAT) score of ≥1. Notably, HMB was more prevalent in women with a fibrinolytic disorder (94%) than in women with a coagulation factor deficiency (74%). However, there was no relationship between the levels of coagulation factor and complaints of HMB. Even women with only mildly reduced coagulation factor activity levels often had HMB.

### Fibrinolytic Disorders in HMB

Fibrinolytic disorders, although rare, are associated with deficiencies or excessive activation of the fibrinolytic system. This can result in severe, lifelong bleeding disorders. The most severe clinical phenotype is caused by α2-Antiplasmin (α2-AP) deficiency. Another bleeding disorder caused by a defect in the fibrinolytic pathway results from Plasminogen Activator Inhibitor-1 (PAI-1) deficiency. This deficiency enhances fibrinolysis due to reduced inhibition of plasminogen activators, resulting in increased conversion of plasminogen to plasmin.^63^ Maas et al^62^ reported that almost all women with a fibrinolytic disorder have complaints of HMB (94%), with HMB defined as a HMB-specific ISTH BAT score ≥1.

### Bleeding Disorders of Unknown Cause (BDUC) in HMB

Bleeding disorders of unknown cause, also classified as unclassified bleeding disorders (UBD), are defined as a clear bleeding tendency in the presence of normal hemostatic tests.^64^ BDUC patients are predominantly female (>60%), with symptoms like HMB and postpartum hemorrhage likely contributing to a higher rate of diagnosis in women than in men. No data are available on how many women with BDUC experience HMB, nor on the frequency of BDUC diagnoses in individuals with HMB.

## DIAGNOSING BLEEDING DISORDERS IN HMB

Women presenting with HMB and possibly an underlying bleeding disorder may present to health care professionals in a variety of ways. Many women affected by HMB do not seek medical help, contributing to the continued underdiagnosis and poor treatment.^1^ Typically, women are first seen by their general practitioner, where a limited investigation into HMB is conducted. In the Netherlands, studies on the incidence of HMB in primary care indicate rates ranging from 5.2 to 7 per 1000 women annually.^65^ A large Dutch retrospective 10-year cohort shows a mean annual incidence of HMB of 9.3 per 1000 women of reproductive age, with 18% being referred to a gynecologist.^66^ However, another recent Dutch study shows that few adolescents visit their general practitioner with HMB despite its high self-reported incidence.^67^ General practitioners commonly refer women to secondary care, namely gynecologists or hematologists.^32^ In secondary care gynecology services, HMB accounts for 20% of visits.^68^

Diagnosing a bleeding disorder is not an easy task and needs specific expertise. In the workup of bleeding disorders, taking a bleeding and family history is crucial to discriminate normal from abnormal blood loss. The clinical appreciation of an overview of all bleeding symptoms, next to menstrual bleeding, is a fundamental step in the evaluation of patients for a possible bleeding disorder. Standard coagulation laboratory tests are not able to identify all common bleeding disorders. Due to inconclusive and expensive laboratory tests, it can be difficult to diagnose or definitely rule out bleeding disorders.^69^ Therefore, it is necessary to combine and use the expertise of both gynecologists and hematologists.

### Bleeding Assessment Tools in HMB

There are several international guidelines for evaluating a (suspected) bleeding disorder in HMB patients. The National Institute for Health and Care Excellence (NICE) recommends to consider testing for coagulation disorders in women who have HMB since the start of their period, and a personal or family history suggesting a coagulation disorder.^21^ The American College of Obstetricians and Gynecologists (ACOG) offers a committee opinion (2019) on screening adolescents who present with HMB; evaluation should include assessment for the presence of anemia from blood loss, including serum ferritin, the presence of an endocrine disorder leading to anovulation, and evaluation for the presence of a bleeding disorder. The obstetrician-gynecologist should be aware of risk factors and comorbidities associated with bleeding disorders.^70^ This committee furthermore recommends that adolescents who fulfill the criteria of the adapted Philipp tool (2011)^71^ (see Philipp tool section) should undergo laboratory testing to screen for a bleeding disorder. If an obstetrician-gynecologist suspects a bleeding disorder in an adolescent patient, collaboration with a hematologist is advised for further laboratory evaluation and treatment planning.^22^

Various instruments are available to help health care providers to distinguish which individuals with HMB require further hemostatic testing for bleeding disorders. Some of these tools have known diagnostic accuracy, while others have not yet been evaluated. Despite these differences, these instruments can help to identify HMB patients who may need further coagulation testing.

#### PBAC

The PBAC is a self-assessed and validated tool developed to visually assess the amount of menstrual blood loss using saturation of sanitary products as a guide [Supplementary material 1, Supplemental Digital Content 1, http://links.lww.com/OBGYNSURV/A43]. By counting the sanitary products used in a period, a total point score can be calculated. The PBAC is a particularly effective tool for identifying abnormal menstrual bleeding when the score is 100 or higher.^16^ This threshold is also a strong indicator of a potential bleeding disorder in patients with idiopathic HMB.^72,73^ A menstrual duration of >5 days is less effective in detecting an underlying bleeding disorder compared with a PBAC score of 100 or more.^73^ A PBAC score >100 was shown to be profoundly more common in women with bleeding disorders such as von Willebrand disease (74%), hemophilia carriers (57%), and FXI deficiency (59%), compared with control groups (29%).^72^ However, while the PBAC is a useful tool for assessing menstrual bleeding, it is not specifically designed and validated to identify individuals who may need further evaluation for bleeding disorders.

#### Philipp Tool

In 2008, Philipp and colleagues developed a screening tool to identify HMB patients requiring hemostatic testing [Supplementary material 2, Supplemental Digital Content 1, http://links.lww.com/OBGYNSURV/A43]. This tool was based on a single-center study involving patients aged 13 to 53 years with HMB unrelated to structural abnormalities.^74^ The tool consists of a questionnaire with 8 questions across 4 categories. It is considered positive if at least one of the following 4 criteria is met: (1) the duration of menses was ≥7 days and the woman reported either “flooding” or impairment of daily activities during most periods, (2) a history of treatment of anemia, (3) a family history of a diagnosed bleeding disorder, or (4) a history of excessive bleeding with tooth extraction, delivery or miscarriage, or surgery. A positive result indicates that the patient should undergo laboratory screening for a potential bleeding disorder. The study reported a sensitivity of 82% (95% CI: 75-90) for detecting bleeding disorders. Including a PBAC with a cutoff score >100 increased the sensitivity of this Philipp screening tool to 94% (95% CI: 89 to 99). Separate subanalysis of adolescents (≤19 y) and adults (20 to 44 and ≥45 y) did not significantly affect the sensitivity of the combined screening tool with the PBAC score for bleeding disorders (92% and 95%, respectively). The specificity of the screening tool was low (24%, 95% CI: 12-37) and remained low with the addition of the PBAC score >100 (16%, 95% CI: 5-26). Since the tool was designed to stratify women for comprehensive hemostatic testing rather than as a diagnostic instrument, high sensitivity was preferred over specificity. In a multisite follow-up study of women with HMB (ages 18 to 50), the screening tool’s effectiveness for detecting bleeding disorders was further evaluated. Several adaptations were made to the original questionnaire.^71^ The tool then showed 89% sensitivity and 16% specificity for detecting hemostatic abnormalities. When combined with a PBAC score >185, the sensitivity increased to 95%, though specificity dropped to 6%. This indicates the Philipp tool with a PBAC score >100 provides the best balance between sensitivity and specificity. Together, these studies highlight the tool’s strength in ensuring that cases are not missed, but underscores the need for more specific diagnostic follow-ups. While beneficial for capturing potential bleeding disorders, this low specificity also means more referrals. This may increase the workload and resource use at hematology departments.

In 2018, Zia and colleagues determined the diagnostic accuracy of this tool in adolescents with HMB presenting to a secondary care setting. They concluded that although the Philipp tool was sensitive, it poorly distinguished between adolescents with and without hemostatic defects in the context of HMB.^75^ The authors recommend that comprehensive hemostatic testing remains essential to rule out bleeding disorders in adolescents with significant HMB. They moreover underscore the need for adolescent-specific HMB tools in secondary care.

#### Set of Symptoms and Signs

In 2021, Luiro and Holopainen^76^ published a table outlining clinical signs and symptoms indicative of HMB and an underlying bleeding disorder. They recommend further evaluation for a bleeding disorder in adolescents with menorrhagia who also have additional signs of a bleeding disorder or a family history of a bleeding disorder. These screening criteria for adolescents with HMB have, however, not yet been evaluated for diagnostic accuracy.

Curry and colleagues also published a table outlining symptoms and signs suggestive of an inherited bleeding disorder as a cause for HMB. This table is intended to serve as a “prescreening” before using the bleeding assessment tool (BAT) advised by The International Society on Thrombosis and Haemostasis (ISTH).^32^ The authors recommend inquiring about key factors such as the timing (HMB since menarche) and severity of HMB (necessitating hospital admission, requiring blood transfusion, unresponsive to conventional therapy), bleeding history, family history, and physical signs of hypermobility. If there is a strong suspicion of an underlying bleeding disorder, they recommend proceeding with the ISTH BAT. These key screening criteria from the table have yet to be assessed for their diagnostic accuracy.

#### ISTH-BAT

The ISTH BAT was developed by the ISTH as a standardized tool for bleeding symptom measurement in 2010.^77^ This validated BAT is the most commonly used tool for distinguishing between normal and abnormal bleeding and should be performed by a trained specialist. This physician-administered questionnaire includes 14 domains and assigns a numerical score to various bleeding symptoms based on their severity. It includes epistaxis, surgery-related bleeding, menstrual bleeding, and bleeding during childbirth. Normally, a cutoff score ≥6 (with a normal range of 0 to 5) in adult women is used as a predictor for an underlying bleeding disorder.^78^ However, in populations suffering from HMB, especially adolescents, a lower cutoff score appears more optimal in predicting a bleeding disorder. Zia et al^46^ advise using an ISTH-BAT score ≥4 as a predictor of a bleeding disorder in adolescents (10 to 19 years old) with HMB (OR: 8.27; 95% CI: 2.60, 26.44). Öner et al^79^ demonstrated that an ISTH-BAT score of ≤3.5 accurately excludes bleeding disorders in adolescents (10 to 18 years old) with HMB. In addition, Jain et al^80^ analyzed 200 adolescents (postmenarchal—21 years old) with HMB, revealing that an ISTH-BAT cutoff of >4 is fairly specific for identifying bleeding disorders in this group. Notably, Doherty and colleagues found that ISTH BAT menorrhagia domain scores varied considerably with age. For women aged 18 to 30 years, an ISTH-BAT cutoff of ≥5 is recommended (normal range 0 to 4). For women over 30 years, an ISTH BAT score of ≥6 suggests a potential underlying bleeding disorder (normal range 0 to 5 for 31 to 41 years and 0 to 6 for 42 to 51 years).^81^ The variability in cutoff values reported in the literature highlights the need for further refinement of the ISTH-BAT, with age-adjusted reference ranges, to improve its sensitivity and specificity among females. One study used the ISTH-BAT to explore its association with outcomes of endometrial ablation (EA) in women with HMB.^82^ An ISTH-BAT score of ≥6 was linked to younger age at EA, lower rates of amenorrhea, and a higher incidence of dysmenorrhea post-EA. These findings could support more personalized gynecological counseling and treatment for women with elevated ISTH-BAT scores.

#### Self-BAT

Since the ISTH-BAT questionnaire is relatively time-consuming to complete during consultation and requires specialized expertise, a self-administered version was developed. This version is called the self-BAT[Supplementary material 3, Supplemental Digital Content 1, http://links.lww.com/OBGYNSURV/A43]. It was optimized and validated for von Willebrand disease in 2015, achieving a sensitivity of 78% and a specificity of 23% using an abnormal bleeding score defined as ≥6 in females.^83^ This bleeding assessment tool allows patients to complete the questionnaire without assistance, in preparation for the consultation. This saves valuable time for clinicians during outpatient visits.

This study demonstrated that the final optimized version of the self-BAT can generate bleeding scores comparable to those of the expert-administered ISTH-BAT. The authors concluded that it is a reliable and effective screening tool. It is particularly useful for assessing individuals, especially women being assessed for VWD.^83^ Due to the prevalence of VWD, this study centered on that diagnosis. However, additional studies are required to assess the self-BAT’s effectiveness as a screening tool for other hemostatic disorders. In line with this, in 2019, the self-BAT was further validated for detecting bleeding tendencies in patients being evaluated for congenital platelet disorders.^69^ Punt and colleagues recommend the self-BAT as a sufficiently reliable and feasible tool for detecting bleeding tendencies in patients with a (suspected) congenital platelet disorder. They found that the optimal cutoff values for the self-BAT in adults were the same as for the original ISTH BAT.^69^ They reported a sensitivity of 97.1% and a specificity of 43.8% for women at a cutoff of ≥6 (normal range 0 to 5).^69^ While they advise using the self-BAT in clinics, they emphasize that results should be carefully interpreted during consultation. They also call for further studies in primary and secondary care to assess their broader screening potential for bleeding disorders. The Danish study group identified an abnormal self-BAT score of ≥5 in adolescent girls (ages 12 to 17 years) and ≥9 for adult women, although these cutoffs reflect the general population. They recommend further studies to establish the accuracy of these cutoffs.^84^ Interestingly, Eising et al^85^ found that 68% of adult HMB patients who returned the self-BAT had an abnormal total self-BAT score, wherein they used a cutoff score of ≥5 for women aged 18 to 30 years, and ≥6 for women over 30 years. However, completing the self-BAT (which contains a minimum of 13 and a maximum of 60 items) can be a burden for patients, as evidenced by this cross-sectional survey in which 64% of HMB patients did not return the questionnaire (although no reminders were sent). To date, the combined use of the self-BAT and PBAC as an approach for identifying females with HMB who may require evaluation for bleeding disorders has not been studied. Thus, data on its sensitivity, specificity, and feasibility are unavailable. While the self-BAT is not frequently used in the HMB population, a recent study used the self-BAT to measure menstrual health in adolescents and young adults with sickle cell disease.^86^ This study demonstrates its potential applicability in diverse clinical scenarios. In addition, Eising et al^85^ found that total self-BAT scores were associated with the choice of treatment for HMB. They conclude that a bleeding tendency may influence the choice for a hysterectomy over more minimally invasive treatments.

### Comprehensive Hemostatic Testing

Several guidelines offer recommendations for laboratory testing in cases of HMB suspected to be caused by an underlying bleeding disorder.^21,22^ Anemia should be assessed including serum ferritin in all adolescents with HMB.^22^ The NICE guidelines recommend considering testing for bleeding disorders, such as von Willebrand disease, in women who have experienced HMB since menarche and have a personal or family history indicative of a bleeding disorder. They do not specify the tests to be performed.^21^

Curry et al^32^ have recently designed a flowchart setting out appropriate investigations for women with HMB who are considered at risk for a bleeding disorder. This flowchart advises gynecologists to perform at least a full blood count, ferritin and/or iron studies, and to refer patients to the hematology department while awaiting results. Although, iron deficiency caused by HMB is common in women with known bleeding disorders,^87,88^ serum ferritin testing is not included in the ISTH algorithm for diagnosing any bleeding disorder.^89^

The ACOG Committee on adolescent health care recommends that the evaluation of adolescent girls who present with HMB should include assessment for anemia due to blood loss, including serum ferritin, the presence of an endocrine disorder leading to anovulation, and evaluation for the presence of a bleeding disorder.^22^ The Dutch Society of Obstetrics and Gynecology recommends to measure hemoglobin levels at least once in women presenting with complaints of HMB, without mentioning suspicion of an underlying bleeding disorder.^20^ However, a normal hemoglobin level does not rule out the possibility of HMB.^90^ Testing for a hemostatic defect to diagnose an underlying bleeding disorder is mandatory if a woman has consistently heavy menses since menarche and a family history of bleeding or personal history of bleeding with other hemostatic challenges.^91^ There is no strict protocol for testing HMB patients with suspicion of an underlying bleeding disorder. No single laboratory test is adequate for screening the entirety of hemostasis. Several tests are available to screen the coagulation system. Commonly used tests include platelet count, prothrombin time (PT) and activated partial thromboplastin time (aPTT). These tests can be endorsed with assessments of fibrinogen activity, von Willebrand factor activity, and factor VIII activity. Additional testing by a hemostasis specialist is needed in case of a high suspicion of a bleeding disorder, even if initial laboratory tests show no abnormalities. Notably, the 2 most common bleeding disorders associated with HMB, von Willebrand disease and platelet disorders, are not routinely picked up with standard coagulation tests.^32^

## RECOMMENDATION HOW TO SCREEN INDIVIDUALS WITH HMB FOR FURTHER HEMOSTATIC TESTING FOR BLEEDING DISORDERS

Considering the abovementioned available instruments and laboratory tests, a practical combination should be offered to gynecologists to effectively distinguish between HMB with or without a suspected underlying bleeding disorder. Assuming that the patient fulfills the definition of HMB, “excessive menstrual blood loss that interferes with a woman’s physical, emotional, social and material quality of life,”^24^ we recommend that gynecologists start the diagnostic process with either the Philipp tool or the self-BAT. In addition, assessing the PBAC score is optional, as a value of 100 or more is a strong indicator of an underlying bleeding disorder.^73^ Completing a PBAC may, however, not always be feasible due to practical constraints. In some cases, there is no full menstrual cycle between scheduling the appointment and the first gynecological consultation. Moreover, if the recent HMB definition^24^ is applied, a PBAC may not be necessary. The PBAC or self-BAT can potentially be completed by the patient before the first consultation.

**FIG. 1:**
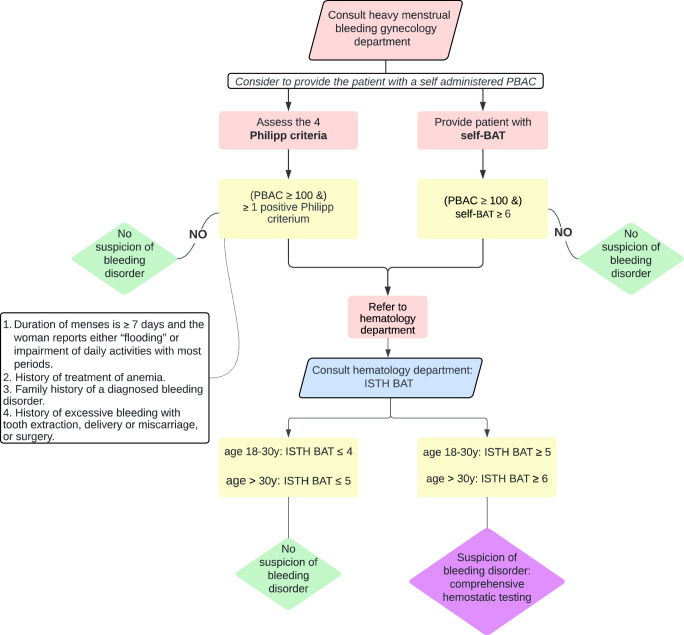
Diagnostic workflow for gynecologists: standardized screening for underlying bleeding disorders in women with HMB.

To alleviate the burden on gynecologists from using the extensive and expertise-dependent ISTH BAT questionnaire, the Philipp screening tool or self-BAT offers a more workable alternative. The Philipp tool’s simplicity, ease of use, quick administration, and specific development for HMB make it a valuable option. Particularly due to its high sensitivity, it ensures that most cases of potential bleeding disorders are identified. The self-BAT, offering similar sensitivity but somewhat higher specificity compared with the Philipp tool, also presents an option. While the self-BAT has not yet been extensively studied across all bleeding disorders, its higher specificity may result in fewer unnecessary referrals. However, it may place a greater burden on the patient, with a risk of incomplete or no responses, while also adding to the gynecologist’s workload for reviewing and interpreting the completed tool. Therefore, the choice between these tools should consider the clinical context, practical considerations, and balancing ease of use, accuracy, and the patient’s role in the process. If HMB is diagnosed or the PBAC is elevated (≥100) and either the Philipp tool is positive, or the self-BAT yields a high score, we recommend referring these women to the hematology department for ISTH BAT administration and further laboratory testing, as illustrated in Figure 1. Based on the literature, we suggest using a cutoff of self-BAT ≥5 for women aged 18 to 30 years, and ≥6 for women over 30 years, for the clinical assessment of bleeding tendency.^69,74,77–79,81,82,84,92^ This approach will effectively identify patients for referral, although it may also result in an increased number of false positive referrals due to the low specificity. These false positives will, however, be ruled out with additional hemostatic testing.

## THE IMPORTANCE OF MORE AWARENESS OF UNDERLYING BLEEDING DISORDERS IN HMB

Significant health risks can occur from undiagnosed bleeding disorders: bleeding with surgery, postpartum hemorrhage, or unnecessary HMB treatment like hysterectomy. An accurate diagnosis is essential to protect women from these potential bleeding risks or unnecessary treatments. We need to raise international awareness for a timely diagnosis and optimal management of bleeding disorders in HMB patients. Therefore, several initiatives have already been undertaken. In 2018, the European Association for Hemophilia and Allied Disorders (EAHAD) founded the Women and Bleeding Disorders Working Group. They performed a large survey among 59 European hemophilia treatment centers, concluding that there is a high priority need for efficacious multidisciplinary treatment for HMB and research into therapies for HMB.^93^ The EAHAD included early recognition and optimal management of HMB, also as 1 of the 10 European principles of care for women with bleeding disorders.^38^ Similarly, the ISTH convened an international expert panel, which assigned the hemostatic management of HMB as appropriate care consideration in bleeding disorders.^94^

## CONCLUSIONS

There is a well-established yet underestimated clinical correlation between bleeding disorders and HMB. While the exact prevalence is unknown, research indicates that 10% to 62% of women presenting with HMB are affected by an underlying bleeding disorder, and screening for an underlying bleeding disorder should be performed. The recognition and screening of bleeding disorders in women presenting with HMB is essential in the evaluation and workup of HMB within the gynecologic field. For now, within the gynecology practice, we advise to use either the Philipp tool or the self-BAT, and if possible in combination with the PBAC, to help distinguish between HMB with or without a suspected underlying bleeding disorder. However, an optimized diagnostic pathway that balances high sensitivity and specificity for systematic screening of bleeding disorders in women with HMB remains to be found. This also aligns with Zia and colleagues' call for future research on optimizing or developing adolescent-specific HMB tools for secondary care settings that aim to balance high sensitivity with improved specificity.^75^ Raising awareness of underlying bleeding disorders is most significant to patients and health care providers, necessitating targeted education and clear guidance. Careful screening and consultation with a hematologist is critical when a bleeding disorder is suspected.

## Supplementary Material
