# Supplementary material for: Heavy Menstrual Bleeding in the Gynecology Clinic: A Call for Awareness and Standardized Screening for Underlying Bleeding Disorders
Source: Obstet Gynecol Surv. 2026 May 7;81(5):241–50. doi: 10.1097/OGX.0000000000001514 (PMC13148511; doi:10.1097/OGX.0000000000001514)
Supplement: Supplementary file 1 [file ogx-81-241-s001.docx]

**Supplementary material 1. Pictorial blood assessment chart (PBAC) for assessment of menstrual blood loss, reprinted with permission from (16)**


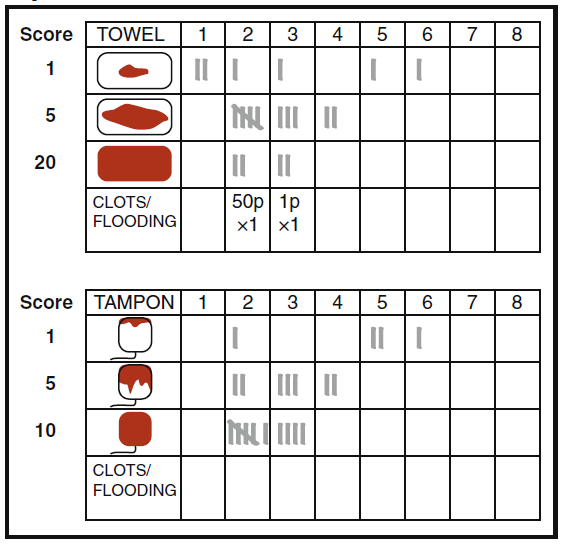


**Supplementary material 2. The adapted Philipp tool for identifying women with HMB requiring hemostatic evaluation for bleeding disorders, reprinted with permission from (71)**

1. How many days did your period usually last, from the time bleeding began until it completely stopped?
   - Less than 7 days
   - Greater than or equal to 7 days
   - Don’t know
2. How often did you experience a sensation of “flooding” or “gushing” during your period?
   - Never, rarely, or some periods
   - Every or most periods
   - Don’t know
3. During your period did you ever have bleeding where you would bleed through a tampon or napkin in 2 hours or less?
   - Never, rarely, or some periods
   - Every or most periods
   - Don’t know
4. Have you ever been treated for anemia?
   - No
   - Yes
   - Don’t know
5. Has anyone in your family ever been diagnosed with a bleeding disorder?
   - i. No
   - Yes
   - Don’t know
6. Have you ever had a tooth extracted or had dental surgery?
   - No (If no, go to question 7)
   - Yes
   - Don’t know

6a. Did you have a problem with bleeding after tooth extraction or dental surgery?

- - No
  - Yes
  - Don’t know

1. Have you ever had surgery other than dental surgery?
   - No (If no, go to question 8)
   - Yes
   - Don’t know

7a.Did you have bleeding problems after surgery?

- - No
  - ii. Yes
  - Don’t know

1. Have you ever been pregnant?
   - i. No
   - Yes
   - Don’t know

8a. Have you ever had a bleeding problem following delivery or after a miscarriage?

- - i. No
  - Yes
  - Don’t know

The screening tool is considered to be positive if 1 of the following 4 criteria were met:

1. The duration of menses was greater than or equal to 7 days and the woman reported either “flooding” or bleeding through a tampon or napkin in 2 hours or less with most periods;
2. A history of treatment of anemia;
3. A family history of a diagnosed bleeding disorder; or
4. A history of excessive bleeding with tooth extraction, delivery or miscarriage, or surgery

**Supplementary material 3. The self-BAT for identifying patients with suspected bleeding disorders, reprinted with permission from (83)**
